# Effect of non-steroidal anti-inflammatory drugs on post-surgical complications against the backdrop of the opioid crisis

**DOI:** 10.1186/s41038-018-0128-x

**Published:** 2018-09-13

**Authors:** Hannah Zhao-Fleming, Audrey Hand, Kelly Zhang, Robert Polak, Armand Northcut, Daron Jacob, Sharmila Dissanaike, Kendra P. Rumbaugh

**Affiliations:** 1 Department of Surgery, Texas Tech University Health Sciences Center, 3601 4th Street, STOP 8312, Lubbock, TX 79430 USA; 2 Burn Center of Excellence, Texas Tech University Health Sciences Center, Lubbock, TX USA; 3 School of Medicine, Texas Tech University Health Sciences Center, Lubbock, TX USA

**Keywords:** NSAIDs, Opioids, Anastomotic leaks, Necrotizing soft tissue infections, Bleeding, Orthopedic injuries, Wound healing, Cancer care, non-steroidal, anti-inflammatory

## Abstract

The USA is currently going through an opioid crisis, associated with tremendous economic and societal impacts. In response to this crisis, healthcare professionals are looking for alternative pain management methods, and non-steroidal anti-inflammatory drugs (NSAIDs) are a sensible choice because of their effectiveness after surgical procedures. However, before surgeons start prescribing NSAIDs in place of opioids, it is crucial to first understand their potential post-surgical complications. The goal of this review is to summarize the data obtained through both animal and human studies, which suggest how a dramatic increase in NSAID use may affect these post-surgical complications. We first provide a short review outlining the mechanisms of action of NSAIDs, followed by a summary of animal studies, which show a trend towards the negative effects of NSAIDs on wound healing and an association between NSAID use and wound infections. Lastly, we present evidence from human studies on the association of NSAIDs with the following complications: anastomotic leaks, necrotizing soft tissue infections, bleeding complications, orthopedic injuries, wound healing, and cancer care. The human studies are much more variable in their conclusions as to whether NSAIDs are beneficial or not, with the only strong evidence showing that NSAIDs inhibit bone healing. This may partially be explained by male and female differences in response to NSAIDs as many animal studies showing the inhibitory effects of NSAIDs were performed on females, while all the human studies were performed with both sexes. We conclude that strong caution should be used in the prescription of NSAIDs, especially in female patients, but larger scale studies are warranted before solid recommendations can be made.

## Background

The ongoing opioid epidemic in the USA has had ravaging effects on the population. The rate of opioid overdoses has tripled since 2000 [1], and the economic damage of this epidemic is estimated to be $78.5 billion due to costs from health care, criminal justice, and lost productivity [2]. One major cause of this epidemic is the incredibly high number of opioid prescriptions, which has quadrupled since 1999 [1]. Numerous studies describe an association between opioid prescriptions and a progression to chronic opioid dependence [1–5].

A leading source of opioid prescriptions is for post-surgical pain management, which is often the setting for opioid-naive patients to first gain exposure to opioids [3–5]. Between 2004 and 2012, 80% of patients who underwent a low-risk surgical procedure filled a prescription for opioids, and over 85% of those prescriptions were for oxycodone and hydrocodone, the two most common opioids implicated in opioid-related overdose deaths [1, 5]. In an effort to reduce the rate of opioids prescribed, former surgeon general Dr. Vivek Murthy urged physicians to use non-opioid treatments for analgesia [6]. In the context of post-operative pain management, a promising alternative is non-steroidal anti-inflammatory drugs (NSAIDs). Some commonly prescribed NSAIDs and their pharmacology are summarized in Table 1. NSAIDs have been shown to reduce the need for opioids following surgery while simultaneously providing the necessary analgesic effects for acute pain management [7, 8]. However, NSAIDs have the potential to cause non-therapeutic effects. With the current national focus on reducing opioid addiction, it is likely that the use of NSAIDs will dramatically increase. Our objective here is to review the mechanisms of action for NSAIDs, to evaluate their effects on surgical outcomes, and to assess the advantages and disadvantages of using NSAIDs as a non-opioid alternative for post-operative pain management.

**Table 1:** Commonly prescribed NSAIDs and their pharmacology

Enzyme inhibited	Name		Bio-availability (%)	Half-life (h)	Dose (mg)	Dosing interval (h)	Max daily dose (mg)
	Chemical	Trade					
COX-1 and COX-2	Aspirin		50–75	0.33	Enteric coated: 325 500 650	4	4000
	Ibuprofen	Advil Motrin IB	80	2	200	4–6	1200
	Diclofenac potassium	Cambia Cataplase Zipsor	50–55	1–2	25 50	6–12	200
	Indomethacin	Tivorbex	~ 100	7.6	20 40	8–12	200
	Naproxen	Aleve Naprosyn	95	12–17	375 500	6–8	1250
	Piroxicam	Feldene	N/A	50	10 20	24	20
	Etodolac	Lodine	80	Tablet: 6.4	Tablet: 400 500	6–8	1200
	*Ketorolac tromethamine	Toradol	100	5.2–5.6	10	4–6	Age 17–65: 120 Age 65+: 60 Max 5 days
COX-2 selective	Celecoxib	Celebrex	N/A	11	50 100 200 400	12	800
	**Nimesulide		54–64	1.8–4.7	100	12	200 Max 15 days

The values listed are for per oral (PO) formulation unless otherwise indicated. *Intravenous (IV) values shown; PO formula discontinued in the USA. **Not available in the USA. The majority of this table was compiled based on two online databases [57, 58], except data for nimesulide [59]. *COX* cyclooxygenase

## Review

### NSAID mechanisms of action

NSAIDs provide anti-inflammatory, antipyretic, analgesic and thrombotic effects through the inhibition of the enzymes cyclooxygenases 1 and 2 (COX-1 and COX-2). These enzymes play a key role in the synthesis of prostaglandin H_2_ (PGH_2_) from arachidonic acid (AA) [9]. PGH_2_ is a precursor for prostaglandins and thromboxanes, collectively referred to as prostanoids, which are synthesized from PGH_2_ through tissue-specific isomerases [9]. Some biologically active prostanoids include prostaglandin E_2_ (PGE_2_), prostaglandin D_2_ (PGD_2)_, prostacyclin I_2_ (PGI_2_), and thromboxane A_2_ (TXA_2_) [9].

These molecules have diverse functions across a multitude of cell types in vivo. PGE_2_ is a pro-inflammatory prostanoid that acts on the kidney, gastrointestinal (GI) mucosa, and the brain. Within the GI tract, PGE_2_ promotes mucus secretion, production of bicarbonate, and mucosal vasodilation [10]. When PGE_2_ synthesis is inhibited, the GI mucosa is no longer protected from stomach acid, resulting in a higher potential for peptic ulceration and epithelial injury [10]. In the renal system, PGE_2_ acts as a pre-glomerular vasodilator, allowing for sufficient renal perfusion [9]. If PGE_2_ synthesis is limited, the glomerular filtration rate is reduced, leading to reduced renal perfusion, thus creating the possibility for acute kidney injury. PGD_2_ is a prostanoid that is predominantly produced by mast cells and acts on D prostenoid receptors (DP_1_ and DP_2_) [9]. During an allergic response, PGD_2_ mediates a pro-inflammatory response, especially in the lungs, causing vasodilation and bronchoconstriction [9]. PGD_2_ has also been shown to inhibit hair follicle neogenesis [11]. TXA_2_ and PGI_2_ are two prostanoids that play significant roles regulating vasodilation and thrombosis within the cardiovascular system [9]. TXA_2_ production is primarily controlled by COX-1, while PGI_2_ production is mediated by COX-2 [9]. TXA_2_ acts a vasoconstrictor and platelet aggregator, while PGI_2_ has opposite effects, acting as a vasodilator and inhibitor of platelet aggregation [9]. Together, these two prostanoids maintain vascular homeostasis. However, when either COX-1 or COX-2 is inhibited through the use of NSAIDs, vascular complications can arise. When COX-2 is selectively inhibited, PGI_2_ production is reduced and the pro-thrombotic activity of TXA_2_ is left unbalanced [9]. This can lead to thrombotic injuries such as myocardial infarction, stroke, and deep-vein thrombosis. Because of the many pathways in which NSAIDs are involved, caution must be applied when prescribing them to prevent unwanted side effects.

### NSAID animal studies

#### Effects on wound healing

Proper wound healing requires a strict series of events [12, 13]: hemostasis (vascular restriction and blood clotting), inflammation (clears out dead cells and pathogens), proliferation (including re-epithelization, angiogenesis, collagen synthesis, extracellular matrix formation, and wound contraction) [14], and remodeling (collagen remodeling with vascular maturation and regression). NSAIDs can disrupt several processes in the proliferation step through inhibition of the COX pathways [15]. COX-coupled prostaglandins, namely PGE_2_ and PGD_2_, are important in wound healing [16]. NSAID-treated wounds were shown to display decreased keratinocyte proliferation and decreased vascular endothelial growth factor (VEGF) expression. This was confirmed in later studies that demonstrated NSAID-mediated decreases in PGE_2_ reduced VEGF expression in keratinocytes [17]. Reduction in PGE_2_ levels by the COX2-specific inhibitor, celecoxib, has also been shown to impair wound healing [18]. This deficit is facilitated by a decrease in re-epithelialization through impaired keratinocyte proliferation, reduced angiogenesis, and reduced granulation tissue in the wound. Decreased myofibroblast differentiation in the wound, along with less wound contraction, was seen in celecoxib-treated mice. NSAIDs also reduced 12-hydroxyheptadecatrienoic acid (12-HHT) production, a ligand for leukotriene B_2_ receptor 2 (BLT2), inhibiting healing in mouse corneas [19]. As the 12-HHT/BLT2 pathway is present in the skin [19], this suggests the possibility that NSAIDs may inhibit wound healing through inhibition of this pathway post-skin trauma.

NSAIDs are also involved in reducing levels of inducible nitric oxide synthase (iNOS) [20]. However, the effect of reduced iNOS on wound healing is controversial. Studies have shown a decrease in iNOS inhibits wound healing because of impaired re-epithelialization, specifically impaired keratinocyte proliferation [21]. On the other hand, a celecoxib-induced decrease in iNOS has been shown to increase re-epithelialization through increased myofibroblast proliferation, increased collagen deposition, and decreased skin necrosis [22]. However, the same group showed that this reduction in iNOS caused decreased angiogenesis and decreased tenascin C, which is important in regulating cell proliferation during embryonic development and wound healing [23].

Interestingly, sex differences in response to NSAIDs have also been reported [24], although the data are somewhat inconsistent. While one study showed that female mice, but not males, displayed delayed healing through decreased collagen formation as a result of aspirin use [24], another demonstrated that female mice did not exhibit decreased healing with a selective COX-2 inhibitor [25]. Paradoxically, this research group also reported an increase in VEGF expression in aspirin-treated female mice. While the majority of studies reported thus far have only utilized female mice, a study using male rats found that, although there were fewer fibroblasts in wounds following NSAID use, wound healing was not delayed [26]. Despite these contrasting studies, animal study evidence supporting a causal relationship between NSAIDs and delayed wound healing exists.

#### Effects on wound infection

NSAIDs have been implicated in increasing the attachment of opportunistic bacteria, such as group A *Streptococcus pyogenes* (GAS), to muscle tissue. The clinical association between NSAIDs and GAS has been previously studied and reviewed in detail [27], yet the molecular mechanisms of this association are not yet completely understood. There is evidence that the protein vimentin is transiently expressed on injured muscle post non-traumatic injury, such as in a strain with eccentric contraction [28]. NSAIDs facilitated GAS attachment to the injured muscle and have been hypothesized to slow muscle regeneration, allowing vimentin to be expressed for longer, increasing GAS attachment. The same group later showed that non-selective NSAID administration increased mortality in mice post-GAS intramuscular challenge [29] and that these non-selective NSAIDs reduced antibiotic efficacy, namely by penicillin and clindamycin. However, they also saw that neither COX-1 nor COX-2 selective NSAIDs made a difference in mouse mortality or antibiotic efficacy, suggesting that nonselective COX inhibitors may have yet unknown effects through other pathways, and that selective COX inhibitors should be favored in patient groups at high risk for infections. Other groups have also shown this association. Weng et al. [30] reported that NSAID administration increased wound area of a GAS intramuscular challenge and increased mortality (100% survival in control groups versus 27.5% survival in NSAID group). Upon histological and molecular analysis, they showed that NSAIDs augmented neutrophilic infiltration into the wound and increased interleukin (IL)-6 and tumor necrosis factor (TNF)-α (both pro-inflammatory cytokines) levels, resulting in greater collateral damage to the host.

Overall, the studies on the direct effects of NSAIDs on wound healing are inconclusive with a skew towards inhibiting wound healing. Results are summarized in Table 2. While there is convincing evidence that NSAIDs inhibit wound healing, there is also evidence that they either have no effect or may even have a beneficial effect by lowering inflammation. However, the effects of NSAIDs cannot be considered in isolation, as perfectly controlled laboratory conditions rarely reflect clinical conditions. The effects of NSAIDs need to be considered in the context of infectious diseases, where NSAIDs may be promoting certain infections by facilitating bacterial attachment to host cells.

**Table 2:** Summary of cited studies on non-steroidal anti-inflammatory drugs

Study	Population	# of patients	Male/female	Pathology	NSAIDs studied	NSAID effect
Kempfer et al. 2003 [16]	Mice	N/A	Female	Wound healing	SC-560 (COX-1 inhibitor), diclofenac, and celecoxib (COX-2 inhibitor)	SC-560 and diclofenac, but not celecoxib, impaired wound repair
Goren et al. 2015 [17]	Mice	N/A	Female	Acute wound healing	SC-560, diclofenac, and celecoxib	Diclofenac impaired wound healing by reducing blood vessel formation
Fairweather et al. 2015 [18]	Mice	N/A	Female	Cutaneous wound healing	Celecoxib	Delayed wound healing through reduced PGE_2_
Iwamoto et al. 2017 [19]	Mice	N/A	Female and male	Corneal wound healing	Diclofenac	Delayed wound healing through inhibition of 12-HHT/BLT2 pathway
Romana-Sousa et al. 2016 [22]	Mice	N/A	Male	Pressure ulcers	Celecoxib	Improved wound healing through decreased iNOS
dos Santos et al. 2013 [24]	Mice	N/A	Female and male	Cutaneous wound healing	Aspirin	Impaired female, but not male, wound healing
Blomme et al. 2003 [25]	Mice	N/A	Female	Surgical skin wound healing	SC-791 (COX-2 inhibitor), diclofenac	No effect
Krischak et al. 2007 [26]	Rats	N/A	Male	Incisional wound healing	Diclofenac	Decreased fibroblast proliferation, but no effect on wound healing
Hamilton et al. 2008 [28]	Mice	N/A	Female	GAS infection following muscle injury	Toradol	Increased GAS in injured muscles
Hamilton et al. 2014 [29]	Mice	N/A	Female	GAS soft tissue infection	Toradol, ibuprofen, indomethacin, SC-560, SC-236 (COX-2 inhibitor)	Nonselective inhibitors reduced antibiotic efficacy and increased mortality. Selective inhibitors had no effect
Weng et al. 2011 [30]	Mice	N/A	Female	GAS soft tissue infection	Ibuprofen	Increased GAS infection and mortality
Kelley et al. 2016 [49]	Humans	443	Not found	Wound healing	Ibuprofen	Ibuprofen does not increase bleeding
Zura et al. 2016 [45]	Humans	309,330	57.9% women	Bone healing	Various	NSAIDs increased non-union
Sagi et al. 2014 [46]	Humans	98	27.6% women	Bone healing	Indomethacin	Indomethacin may increase non-union or decrease heterotropic ossification
Jeffcoach et al. 2014 [47]	Humans	1901	44% women	Bone healing	Various	NSAIDs increase malunion/non-union and infection in long bone fractures
Depeter et al. 2017 [48]	Humans (children 6 months–17 years)	808	37% women	Bone healing	Ibuprofen	Children with extremity fracture do not demonstrate inhibited healing with ibuprofen
Lisboa et al. 2017 [50]	Humans (combat-related extremity wounds)	73	Not found	Wound healing	Various	NSAIDs decreased inflammation and help wound healing
Nagano et al. 2016 [44]	Mouse MC3T3-E1 Cell line	N/A	Not found	Bone formation and fracture healing	Celecoxib	Celecoxib inhibited osteoblast maturation by suppressing Wnt signaling
Smith et al. 2016 [33]	Humans	657	Not found	Anastomic leaks	Various	Post-operative nonselective NSAID use, but not COX 2 inhibitors, increased anastomic dehiscence
Paulasir et al. 2015 [34]	Humans	4360	56.7% women	Anastomic leaks	Various	No difference
Subendran et al. 2014 [35]	Humans	262	45% women	Anastomic leaks	Various	Non-significant increase in anastomic leaks (*p* = 0.06)
Saleh et al. 2014 [36]	Humans	731	43% women	Anastomic leaks	Toradol	No difference
Mikaeloff et al. 2008 [37]	Humans (primary varicella or zoster diabnosis)	248,368	48% women—varicella patients; 61% women—zoster patients	Complications of varicella or zoster infection	Various	Elevated risk of severe skin and soft tissue complications
Klein et al. 2012 [43]	Humans	162	80% women	Bleeding after laparoscopic Roux-en-y bypass	Toradol	Lower post op hemoglobin
Flossmann et al. 2007 [53]	Humans	7588	Female and male	Colorectal cancer	Aspirin	Daily dose aspirin is effective in primary prevention of colorectal cancer
Huang et al. 2018 [55]	Humans (who had surgery for non-small-cell lung cancer)	588	Female and male	Survival length post-surgery	Flurbiprofen axetil	The use of flurbiprofen axetil combined with dexamethasone was associated with longer survival
Yin et al. 2018 [56]	Humans (with bone metastasis)	210	Female and male	Levels of interleukin-6, prostacyclin, corticosteroid A2	Flurbiprofen	Flurbiprofen lowered levels of prostacyclin and corticosteroid A2, but did not affect interleukin-6

Cited studies are summarized. Clinical studies are on adult patients unless otherwise specified. In animal studies, if the study included male and female animals, they are age-matched and equal in ratio. In clinical studies, the male/female ratios are presented as % women if the information was given in the paper. Specific non-steroidal anti-inflammatory drugs (NSAID) names are given when supplied by the authors and are non-selective cyclooxygenase (COX) inhibitors unless otherwise specified at first mention in the table

### NSAID human studies

#### Effects on anastomotic leaks

In colorectal surgery, the institution of enhanced recovery after surgery (ERAS) pathway has led clinicians away from opioid-directed pain control to using acetaminophen and NSAIDs. ERAS decreases opioid requirements by utilizing a multimodal pain approach, leading to less post-operative ileus and a decreased length of stay [31]. The use of NSAIDs has been shown to increase patient satisfaction and minimize adverse events as a consequence of opiate use [32]. With the acceptance of this as standard protocol in many hospitals, such as in our institution, it propagates curiosity if the increased use of NSAIDs will result in a higher number of anastomotic leaks.

Anastomotic leaks in intestinal surgery remain associated with substantial morbidity and mortality. Multiple studies have been conducted to assess the risk of anastomotic leaks as the result of NSAID use, and controversy exists regarding whether this association is of clinical significance. A recent meta-analysis pooled randomized controlled trials and observational studies to determine the strength of association between anastomotic leaks of the small bowel, colon, rectum, and anus with NSAID use [33]. It was reported in 2016, after incorporating the results of all known human studies on anastomotic dehiscence of the intestine and post-operative NSAID use, that the odds of developing an anastomotic dehiscence were approximately one and a half times higher [33]. The previous year, a population of 4360 patients undergoing colorectal anastomoses, pooled from the Michigan Surgical Quality Collaborative (MSQC) database, were analyzed. Twenty-eight percent (1297) of patients received NSAIDs post-operatively while 70% were not given NSAIDs [34]. Both intraperitoneal and extraperitoneal anastomotic leaks were compared. A significant statistical relationship between the use of NSAIDs and the development of an anastomotic leak was not demonstrated [34].

A similar discordance was seen in 2014, when a study out of Mount Sinai Hospital in Toronto prospectively collected data on patients (*N* = 262) undergoing elective colorectal surgery for cancer (34%) or inflammatory bowel disease (66%) [35]. They reported a significantly higher risk of anastomotic leak associated with NSAID utilization [35]. In contrast, the University Health Network in Toronto published contrasting results, where a retrospective review was performed over the course of 7 years, and patients undergoing colon resection with primary anastomosis were identified (*N* = 731) [36]. After adjusting for comorbid conditions such as age, smoking, and steroid use, there was no statistically significant relationship between Toradol use and anastomotic leakage [36]. Overall, further research is needed to clarify the clinical relationship between NSAID use and the risk of developing an anastomotic leak. Widespread utilization of ERAS protocols for colorectal and other intestinal operations affords the opportunity for this to be studied prospectively at multiple centers.

#### Effects on necrotizing soft tissue infections

Necrotizing soft tissue infections (NSTIs) represent a wide spectrum of pathology involving necrosis of the skin, subcutaneous fat, superficial/deep fascia, and muscle that may result in significant morbidity and mortality. Treatment involves aggressive source control with surgical debridement and broad-spectrum antibiotics, and prevention is crucial to decrease the morbidity associated with the disease. NSAIDs have been reported to contribute to the development of NSTIs; however, the association between NSTIs and NSAIDs remains debated. Furthermore, it is difficult to study the relationship between NSAIDs and NSTIs, as NSTIs are uncommon occurrences.

Several studies have reported an association between the development of soft tissue infections in children diagnosed with varicella zoster virus (VZV) and NSAID use. Three epidemiological studies observed an increased risk of invasive GAS infection in children who received NSAIDs when diagnosed with VZV [37]. A case-control study was performed utilizing the General Practice Research Database (GPRD) [37], and all soft tissue infections occurring within a 2-month period after VZV diagnosis were investigated. NSAID prescriptions during the follow-up period were associated with an increased risk of soft tissue and skin infections [37]. Similar results were reported from Toulous University Hospital in France when38 cases of NSTIs were match-controlled and found to have a strong association between NSTIs, NSAID use, and VZV [38].

There are several case studies and series in the literature showing a relationship between NSAID administration and development of NSTIs [37, 39–41]. However, it is difficult to discern causation from correlation. NSAIDs are commonly taken to treat the symptoms associated with NSTI pathology while opioids are not as readily available. Therefore, it is possible that if they were similarly accessible, the same relationship might be apparent.

#### Effects on bleeding complications

The use of NSAIDs has long been known to have effects on coagulation and bleeding. As described above, NSAIDs inhibit COX-1 and COX-2 enzymes. By inhibiting COX-1 enzymes, there is a decrease in TXA_2_ which decreases vasoconstriction and platelet aggregation and can increase bleeding time. When using NSAIDs, the duration of the platelet inhibition and vasoconstrictive effects is based largely on the specific drug’s half-life. The endothelial cells that line the blood vessels serve as a barrier between membranes and have several antithrombotic properties that help maintain normal blood viscosity. For example, ibuprofen has an inhibitory platelet aggregation effect within 2 h and the effects are lost after approximately 12 h [42]. Aspirin, on the other hand, has maximal effects on platelet function within 2 h and can persist for up to 7 days after the dose was taken. Within 12 h of taking a single dose of 325 mg aspirin, bleeding times can sometimes double from baseline values.

As demonstrated, use of NSAIDs can cause undesired blood loss. Clinically, this can become significant in certain populations, including the post-operative patient. The most common site of spontaneous bleeding is the GI tract because of the inhibition of mucosal prostaglandin production, impaired mucosal repair and healing, and the strong interaction with *Helicobacter pylori* [42]. In a study by Schafer et al. patients that underwent hip arthroplasty had more post-operative bleeding complications than patients that did not take NSAIDs in the perioperative period [42].

Patients that have an underlying coagulopathy, including von Willebrand’s disease, thrombocytopenia, or other coagulation factor deficiencies, have exaggerated NSAID- and aspirin-related bleeding times. Patients that have alcohol in their system, or have a history of alcoholism with significant liver impairment, should also be monitored closely for post-operative bleeding. Providers should be hesitant to prescribe NSAIDs or aspirin to these patients in the perioperative period due to their increased risks for prolonged bleeding time and spontaneous bleeding. There are procedures that require special attention to NSAID use in the perioperative period, including cardiovascular surgery, oral cavity manipulation, and genitourinary procedures, because these procedures have increased bleeding tendencies that can be exacerbated by NSAID or aspirin use.

Bleeding in the perioperative period is a multifactorial problem. Klein et al. showed an increased risk of intraoperative bleeding and post-operative bleeding after ketorolac use in patients undergoing laparoscopic Roux-en-Y gastric bypass (7.6% vs 6.4%) in a retrospective review [43]. NSAIDs and aspirin are known culprits of bleeding and increased bleeding tendencies, but there are neither enough prospective nor retrospective studies showing a direct correlation between NSAID use and perioperative hemorrhage.

#### Effects on orthopedic injuries

NSAIDs have long been shunned in the world of orthopedic surgery for their presumed role in the impedance of bony and ligamentous healing. Orthopedic literature based on rat models have proposed that the use of NSAIDs will decrease bone healing and increase rates of the non-union of bones [44]. An inception cohort study by Zura et al. concluded that, along with multiple concurrent fractures, open fracture, anticoagulant use, and osteoarthritis with rheumatoid arthritis, prescription NSAID and opioid use increased the rate of non-union across all bone fractures [45]. In a prospective, randomized, double-blinded trial by Sagi et al. looking at acetabular fractures, indomethacin appeared to decrease heterotrophic ossification, but increase the incidence of non-union of the posterior acetabular wall [46]. Likewise, a retrospective cohort study conducted at a level 1 trauma center by Jeffcoach et al. found that, for patients with femur, tibia, and humerus fractures, those who used NSAIDs in the post-operative period had two times the rate of non-union and/or mal-union as those not taking NSAIDs [47]. On the other side of the debate, a retrospective study published by DePeter et al. showed that, for children aged 6 months to 7 years who presented with a fracture of the tibia, femur, humerus, scaphoid, or fifth metatarsus fractures, ibuprofen use was not associated with non-union or delayed union [48]. Overall, these studies show that NSAID use may correlate with the rate of non-union in long-bone fractures. Thus, NSAIDs should be prescribed judiciously in the post-operative period for pain control, and clinicians should bear in mind the possible effects of non-union or mal-union of long bone fractures when prescribing NSAIDs for pain control.

#### Effects on wound healing

A meta-analysis by Kelley et al. [49] concluded that ibuprofen does not increase bleeding during soft tissue surgical procedures. This study also concluded that ibuprofen was comparable to opiate-based pain medication for post-operative pain control. A retrospective study by Lisboa et al. found that, for combat-related extremity wounds, short-term NSAID use was associated with lower concentrations of inflammatory cytokines and superior wound healing, even after controlling for confounding variables such as wound size and smoking [50]. The authors also looked at healing of operative debrided, combat-related extremity wounds and found that short-term NSAID use was associated with decreased inflammatory factors and more successful wound healing [50]. Darby et al. proposed that aspirin might have a beneficial effect in treating chronic wounds due to its ability to inhibit inflammatory pathways that increase cytokine output while increasing anti-inflammatory molecules that are pro-repair and pro-resolution, such as aspirin-triggered lipoxins [15]. Taken together, these properties of aspirin may promote the healing of chronic wounds that may be trapped in an inflammatory state. While there is a dearth of literature on the use of NSAIDs in wound healing, existing literature suggests that NSAID use does not decrease the wound-healing rates of soft tissue wounds. However, more clinical research still should be done on the use of NSAIDs for wound healing and pain control of both acute and chronic wounds.

#### Effects on cancer care

Most of the literature on the role of NSAIDs in cancer has focused on chemoprevention, rather than wound healing [51, 52]. A systematic review of studies involving more than 20,000 patients with colorectal cancer showed a 20–40% risk reduction with the use of aspirin and other NSAIDs, primarily celecoxib [53] Other common cancers such as breast cancer also express COX-2, indicating a potential for chemoprevention in these cases as well; the data is mixed but there is literature to support this premise [54]. The use of NSAIDs perioperatively in cancer surgery has not been as well studied; in one intriguing study, the use of dexamethasone and flurbiprofen axetil perioperatively was associated with improved survival after resection for non-small cell lung cancer [55]. While the potential mechanism of this post-operative action is unknown, it is likely related to modulation of the inflammatory response, specifically on interleukins [56]. It is likely that the potential benefits of NSAID use in inhibiting the growth and spread of cancer cells outweigh the negative impact on wound healing in cancer patients.

## Conclusion

There is strong evidence in animal studies that non-selective NSAIDs generally inhibit wound healing while either COX-1 or COX-2 selective NSAIDs tend to show no effect on wound healing. On the other hand, clinical studies show much more mixed results, with strong evidence of NSAIDs inhibiting healing only in bone fractures. The other disease states summarized in this review had either low numbers of studies found or studies showing no effect of NSAIDs on healing. Interestingly, NSAIDs also seem to provide a chemoprevention role in cancer care. Important to note, the majority of animal studies were conducted only on female animals, but all human studies are done on a mixed, generally evenly distributed ratio, of male and female patients. The evidence of male and female dichotomy in response to NSAID administration represents an important consideration for future studies. Taken together, larger scale studies are needed to understand the exact effects of NSAIDs on healing, and specifically alternate effects on male and female patients are especially understudied.

## Abbreviations

12-HHT: 12-Hydroxyheptadecatrienoid acid
AA: Arachidonic acid
BLT2: Leukotriene B_2_ receptor
COX: Cyclooxygenase
DP: D prostenoid receptors
ERAS: Enhanced recovery after surgery
GAS: Group A *Streptococcus*/*Streptococcus pyogenes*
GI: Gastrointestinal
GPRD: General Practice Research Database
IL: Interleukin
iNOS: Inducible nitric oxide synthase
MSQC: Michigan Surgical Quality Collaborative
NSAIDs: Non-steroidal anti-inflammatory drugs
NSTI: Necrotizing soft tissue infection
PG: Prostaglandin
TNF: Tumor necrosis factor
TXA: Thromboxane
VEGF: Vascular endothelial growth factor
VZV: Varicella zoster virus
